# Psychological Therapies Used for the Reduction of Habitual Cigarette Smoking Cigarette Consumption: A Systematic Review

**DOI:** 10.3390/ijerph21060753

**Published:** 2024-06-09

**Authors:** Sandra-Milena Carrillo-Sierra, Lorena Cárdenas-Cáceres, John Anderson Cadrazco-Urquijo, Angie Natalia Salazar-Gómez, Diego Rivera-Porras, Valmore Bermúdez

**Affiliations:** 1 Universidad Simón Bolívar, Facultad de Ciencias Jurídicas y Sociales, Centro de Investigación en Estudios Fronterizos, Cúcuta 540001, Colombia; l_cardenas1@unisimon.edu.co (L.C.-C.); j_cadrazco@unisimon.edu.co (J.A.C.-U.); a_salazar7@unisimon.edu.co (A.N.S.-G.); diego.rivera@unisimon.edu.co (D.R.-P.); 2 Universidad Simón Bolívar, Facultad de Ciencias de la Salud, Centro de Investigaciones en Ciencias de la Vida, Barranquilla 080001, Colombia; valmore.bermudez@unisimon.edu.co

**Keywords:** psychological therapy, smoking, addiction, nicotine, cognitive behavioral therapy

## Abstract

Globally, there are around 1.3 billion cigarette consumers, indicating it to be the second highest risk factor for early death and morbidity. Meanwhile, psychological therapy offers tools based on its different models and techniques, which can contribute to smoking cessation. In this context, this study gathers scientific evidence to identify psychological therapies that can be used to reduce cigarette consumption. A systematic review of controlled clinical studies was conducted, implementing the PRISMA methodology. Search queries were performed with terms extracted from MESH (Medical Subject Headings) and DECS (Descriptors in Health Sciences). Subsequently, the search was queried in the scientific databases of Medline/PubMed, Cochrane, Scopus, Science Direct, ProQuest, and PsycNet, with subsequent verification of methodological quality using the Joanna Briggs Institute checklists. The selected documents revealed that cognitive behavioral therapy prevails due to its use and effectiveness in seven publications (25%). The cognitive approach with mindfulness therapy is found in 4 publications (14%), the transtheoretical model with motivational therapy in 4 publications (14%), brief psychological therapy in 3 publications (10%), and the remaining 10 documents (37%) correspond with others. Intervention studies refer to cognitive behavioral therapy as the most used in reducing cigarette consumption; in terms of the duration of abstinence, scientific evidence shows beneficial effects with short-term reduction.

## 1. Introduction

Current use of cigarettes and smokeless tobacco is associated with an increased risk of mortality in general, especially in daily smokers [1]. According to Méndez, Alshanqeety, and Warner (2013), if no additional policies are implemented and current smoking initiation and cessation rates persist, global adult smoking prevalence is estimated to be 22.7% in 2020 and 22.0% in 2030, with a total of 872 million smokers [2].

According to the latest World Health Organization (WHO) report, more than 1.3 billion people consume cigarettes worldwide [3,4]. In Switzerland, smoking was the most common modifiable cardiovascular risk factor among young adults hospitalized for acute coronary syndromes, affecting 71.4% of them. Although there has been a significant decrease in active smoking among these patients between 2000 and 2019, it still affected approximately two-thirds of them in 2019 [5].

Smoking is associated with an increased risk of more than 25 preventable diseases, including chronic obstructive pulmonary disease (COPD), coronary artery disease (CAD), and several types of cancer, including lung, laryngeal, mouth, and throat cancers [6]. Smoking is, therefore, directly linked to increased morbidity and mortality, including chronic diseases such as diabetes mellitus and CAD due to atherosclerosis and high blood pressure [7,8]. According to the WHO [9,10], smoking is the second most common cause of premature death, causing around 8,000,000 deaths per year.

Tobacco, being a legal consumer product, contains components that, when burned, generate numerous toxic substances that negatively affect various organs and systems of the human body, impacting the life expectancy of smokers [11]. Tobacco smoke is composed of a gaseous phase and a particulate phase containing more than 4000 identified substances, such as carbon monoxide, nicotine, and tars [12]. During the combustion of cigarettes, substances such as hydrocyanic acid, formic aldehyde, lead, arsenic, and ammonia, radioactive elements such as uranium, benzene, tobacco-specific nitrosamines, polycyclic aromatic hydrocarbons, and acetaldehyde are created, enhancing the addictive effect of nicotine, the main addictive component of tobacco [13].

Carbon monoxide, a common substance in tobacco, impacts oxygenation and blood vessels, while nicotine generates psychological and physical dependence by binding to specific brain receptors [14]. Moreover, tobacco tars contain carcinogens and irritants that damage the respiratory system and other organs, such as polonium-210, a carcinogenic substance present in tobacco smoke due to the absorption of radium-226 by tobacco leaves [15]. Exposure to these harmful substances impacts health depending on the amount, duration and form of exposure, the presence of other chemicals, as well as individual habits and personal characteristics [16].

Another essential component in understanding smoking is nicotine, an essential component extracted from tobacco leaves, considered addictive due to its ability to generate a transient release of endorphins in the brain’s reward circuits via dopamine [17]. This phenomenon produces a momentary feeling of euphoria when it enters the bloodstream, although its comparatively brief effect requires further consumption of the substance to maintain the desired effect [18]. Regular nicotine use affects the central nervous system, influencing learning, the ability to handle stress, and self-control [19]. When cigarette smoke reaches the lungs, nicotine is rapidly absorbed and reaches the brain within seconds, being a determining factor in the addiction process due to its pharmacokinetic properties [20]. This rapid access to the central nervous system makes it difficult to quit smoking immediately, so an evidence-based therapeutic approach is required to manage withdrawal symptoms [21].

Currently, there are different types of smoking cessation therapies, e.g., pharmacotherapy [22], nicotine patch therapy, and interventions from a psychological perspective (cognitive behavioral therapy [CBT] [23], motivational interviewing [24], mindfulness, telephone support, quit lines, online services, and social networks) [25,26].

Various conventional psychological therapies are mentioned in the literature, such as cognitive behavioral therapy, motivational interviewing, and mindfulness. CBT focuses on changing negative thought patterns, providing strategies for adaptive coping with smoking-related thoughts and behaviors [27]. Motivational interviewing, on the other hand, focuses on empathic collaboration and ambivalence, seeking to strengthen intrinsic motivation to quit smoking, recognizing the complexity of the process [28]. Several studies [29,30] showed promising results when using motivational interviewing as a pre-treatment tool for smoking cessation. On the other hand, mindfulness, based on meditation techniques, focuses on increasing awareness of urges to smoke, facilitating the conscious management of these urges by highlighting the importance of the mind–body connection in overcoming tobacco use [31]. Recent research highlights the effectiveness of social networks, helplines, and online services as a means of providing ongoing support [32,33]. Based on the above, the present systematic review aims to compile scientific evidence on the psychological therapeutic actions used in smoking reduction.

## 2. Materials and Methods

The present study was conducted following the PRISMA method as a guide to collect updated information in a transparent manner, revealing key findings about the review [34]. Inclusion and exclusion criteria were established. The scope of the systematic review was established by the AMCPLT strategy to perform a descriptive analysis of the scientific literature, not including a meta-analysis. The acronym corresponds to six components of the research question: (A) Adjective; (M) Measurement; (C) Condition; (P) Population; (L) Place; (T) Time [35].

**Review questions**

What therapeutic actions or psychological treatments are most used for the reduction of habitual cigarette smoking?

Based on the establishment of the questions, the inclusion and exclusion criteria to be considered for the selection of the information were defined, obtained from consultation databases using search equations with descriptors examined in health thesauri to determine the key words (Table 1 and Table 2).

**Inclusion criteria**

As we delved into this review, we maintained certain criteria in mind while selecting studies. Firstly, we prioritized research grounded in randomized controlled trials (RCTs), particularly those incorporating elements of psychological treatments in their interventions. Furthermore, it was essential that participants were adults, over the age of 18, with a history of regular nicotine use. Additionally, we imposed a temporal boundary on our selection, concentrating on studies published within the last five years (2017–2021).

**Exclusion criteria**

In our approach, we also established specific criteria to exclude certain studies from our analysis. Consequently, we eliminated research that was not based on experimental methods and those lacking psychological interventions in their methodology. Additionally, we disregarded publications dated before 2017, those unrelated to smoking habits, and any texts without full access.

**Information sources**

To conduct our information search, we utilized six renowned scientific bibliographic databases: ProQuest, Science Direct, Scopus, Medline/PubMed, the Cochrane Central Register of Controlled Trials, and PsycNet.

**Search strategy**

To accurately identify the studies and formulate the search equations, we meticulously selected the appropriate terms. We utilized descriptors in Health Sciences, such as Medical Subject Headings (MeSH) and DECS (Health Sciences Descriptors), focusing on the following keywords: “Psychological therapy”, “Smoking”, and “Nicotine dependence” (see Table 1).

To construct the search equations effectively, we employed Boolean operators, including “OR”, “AND”, and “NOT”. These operators were skillfully adapted to suit the format of the various databases we utilized (see Table 2).

Subsequently, we initiated our search using the equations crafted for the previously selected databases. It is noteworthy that some databases accommodated a broad array of terms, while others imposed limits on the number of terms at the outset of a search, leading to varied results. After eliminating duplicate publications that failed to meet our eligibility criteria, we independently reviewed the titles and abstracts. During this phase, studies deemed irrelevant based on our inclusion and exclusion criteria were excluded. Any disagreements regarding the suitability of articles were resolved through thorough discussion. Below, you will find a summary of our filtering process, applied to each criterion in the publications, determining the number of studies selected for inclusion (Table 3).

**Data extraction process**

Following the initial selection, we meticulously prepared a record table in Excel, which was independently populated by the authors. In this table, we carefully documented the key elements from each of the selected studies, ensuring a detailed and organized compilation of data.

**Characteristics**

Initially, we embarked on classifying the interventions and the corresponding descriptions of the treatment therapies. In this process, we compared these interventions between the control and experimental groups. This comparison considered various characteristics of the therapies, such as the underlying model, techniques employed (if applicable), and whether the intervention was conducted in groups or individually. Additionally, we investigated specific details regarding the therapy sessions, including the number of sessions, their frequency, and the duration of each session. The effectiveness and benefits of these therapies, the intervention protocol, randomization processes, and the characteristics of the participants were also thoroughly examined.

Furthermore, we identified and documented the qualifications and characteristics of the therapists and outcome evaluators. The follow-up protocols post-intervention and the findings of the studies were also meticulously recorded. In instances where we encountered missing or unclear data, we proactively reached out through email to request additional information, thereby ensuring the comprehensiveness and accuracy of our data collection process.

**Risk of bias assessment of individual studies**

The approach we adopted for assessing the risk of bias in the included studies was grounded in the critical appraisal tools provided by the Joanna Briggs Institute. Specifically, we utilized their checklist for randomized controlled trials to determine the reliability level of each study. This instrument was applied independently to the checklists, ensuring a thorough and unbiased evaluation. Following this individual assessment, a comparison was made through a consensus-building process, as outlined in reference [27].

**Selection and analysis**

The initial phase of our study selection involved a preliminary screening based on inclusion criteria, focusing on characteristics of the population, study type, and publication year. Following this, we embarked on a more detailed process to determine the eligibility of studies. This involved tabulating their characteristics, which allowed for a comprehensive comparison. During this comparison, we rigorously verified each study’s compliance with the inclusion criteria.

This meticulous process was guided by the criteria set forth in the Joanna Briggs Institute checklist for randomized controlled trials. The checklist includes a range of questions covering key aspects of trial design and execution: randomization methods for assigning participants to treatment groups, the blinding of participants, therapists, and outcome evaluators to treatment allocation, ensuring baseline similarity, verifying complete follow-up, and accounting for dropouts, employing appropriate statistical analysis, and evaluating the adequacy of the trial design. Additionally, it includes queries regarding the potential standard deviation in randomized controlled trials, particularly focusing on individual randomization and parallel groups.

In the final stage of study selection, we extracted and collated relevant sections addressing our research question. These data were then systematically integrated into two distinct tables for clarity and ease of reference. The first table, titled “Selected Studies”, presents general information on the selected studies. The second focuses on therapeutic interventions and is titled “Intervention Characteristics”.

**Assessment of publication bias**

This process was performed based on the Joanna Briggs Institute checklist criteria for randomized controlled trials [36].

## 3. Results

### 3.1. Selection of Studies

**Figure 1:**
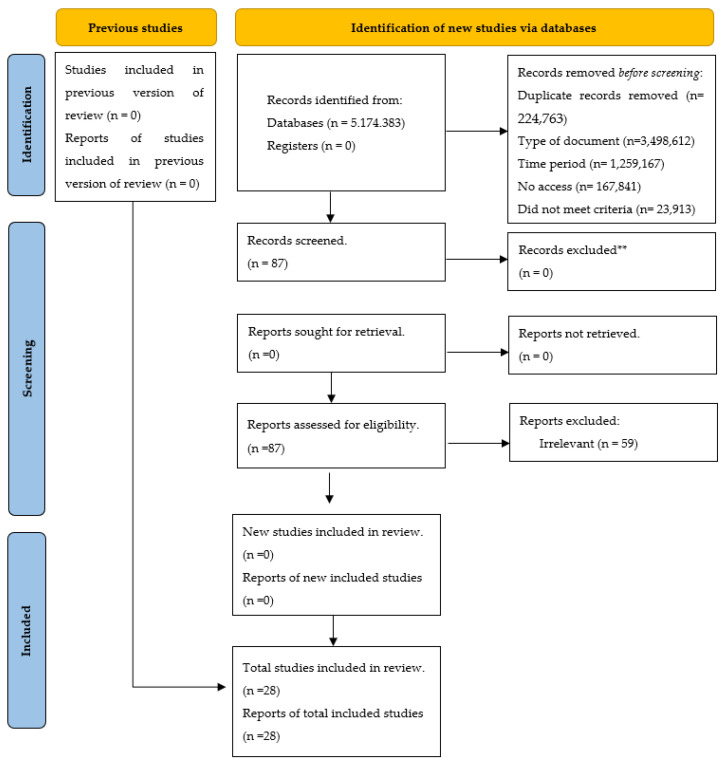
Flow diagram for updated systematic reviews, which includes searches of databases [34].

The results of the search and selection process, from the records found in the review to the inclusion of the studies, are presented below (see Figure 1).

Our search process commenced with the initial step of determining the “Total number of articles found”. This figure represents the aggregate results yielded by the initial search across various databases. At this juncture, we had solely employed the search equation without the integration of any exclusion filters. In addressing the “Document Type”, our focus was narrowed to texts specifically categorized as articles. This delineation led to the exclusion of books, essays, and other such formats, allowing us to concentrate on sources that are more pertinent and specific. The “Time Period” was intentionally confined to the years between 2017 and 2022. The section pertaining to “Incomplete/duplicate texts” covers two significant concerns. Firstly, we omitted texts that were incomplete, whether due to access limitations or their status as ongoing research proposals. Secondly, we removed articles that were either duplicates or had undergone subsequent updates. The category “No access” pertains to articles that preclude the viewing of the complete document. Regarding “Noncompliance with criteria”, texts that failed to meet our predefined exclusion criteria were eliminated. Ultimately, the “Total number of selected articles” indicates the final tally of articles that successfully passed through all filters and criteria and were, thus, deemed suitable for inclusion in our systematic review.

From an initial pool of 23,913 studies, a rigorous process of mechanical filtering was applied, significantly reducing the number of documents. A subsequent detailed review of the texts further narrowed the selection to 28 documents that met our established criteria. Throughout this process, cognitive behavioral therapy (CBT) emerged as a prominent theme, both in terms of quantity and effectiveness, being featured in seven publications (25%). Other notable approaches included the cognitive approach and mindfulness therapy, each found in four publications (14%), as well as the transtheoretical model with motivational therapy. Brief psychological therapy was mentioned in 3 publications (10%), and the remaining 10 papers (37%) explored a variety of interventions, each offering unique insights into the challenge of smoking cessation. During study selection, it was ascertained that CBT is prevalently used due to its efficacy in enhancing adherence to psychological treatments for smoking and in reducing abstinence among habitual cigarette users.

### 3.2. Characteristics of the Studies

As highlighted in the preceding section, our meticulous selection process culminated in the identification of 28 studies (see Table 4).

### 3.3. Risk of Bias of Individual Studies

In assessing the risk of bias in individual studies, the Johanna Briggs checklist, a widely recognized tool for the assessment of randomized controlled trials, was applied. After detailed analysis, most studies were classified as having a medium-low risk of bias.

### 3.4. Analysis of the Treatments

After careful selection and assessment of the risk of bias of each study, a detailed analysis of each paper was conducted. This involved classifying the studies according to descriptions of the interventions and comparisons between treatments and control groups. Specific details of each intervention were examined, such as the number of sessions, their duration and frequency. In addition, we compared how each technique addressed smoking cessation, considering participants and their attributes related to group assignment, methods of measuring smoking, and the presence of pre- and post-testing. The synthesis also included an overview of the therapists and evaluators involved, as well as the follow-up procedures during and after the intervention, and the outcomes of the study, i.e., the efficacy of the therapies.

Of the studies reviewed, seven incorporated cognitive behavioral therapy (CBT) due to its effectiveness in modifying dysfunctional thoughts and behaviors, focusing on the problem and highlighting practical application. In the context of smoking cessation, CBT helps individuals to restructure their thinking patterns, adopt healthier responses, and develop beneficial behaviors. The other studies we analyzed employed a variety of interventions, some of which were combined approaches. For example, Ref. [40] integrated motivational intervention, games, story therapy, and reading and writing therapy. We also encountered mindfulness therapy [45], Allen Carr’s Easyway therapy [41], and cognitive bias modification [62,63].

### 3.5. Techniques Used in the Studies

In this systematic review, we observed a diverse array of intervention techniques across the selected studies, with a notable emphasis on those grounded in the cognitive behavioral therapy (CBT) model. Notably, cognitive restructuring emerged as a key technique, employed in two of the studies. This approach focuses on identifying, confronting, and altering irrational and negative thought patterns that fuel addiction to smoking.

Additionally, we noticed a significant incorporation of various training methods derived from the CBT model. These included self-modification, mindfulness, impulse tolerance training, and emotional regulation. Emotional regulation, which appeared in two of the studies, involves teaching individuals to manage their immediate emotional states—whether that means maintaining, enhancing, or suppressing them—with the goal of achieving emotional equilibrium.

Group counseling sessions, used in two of the studies, provided a platform for participants to build relationships and offer mutual support, aiding them in tackling the everyday challenges associated with smoking. Techniques for stimulus control were also noted in our review.

Motivation played a central role in the implementation of techniques such as motivational interviewing, playful interventions, and motivational interventions, often combined with story therapy, as well as reading and writing therapy. Psychoeducation was identified as technique for effectively managing dependency, withdrawal symptoms, stimulus control, self-regulation, mood management, coping skills, and relapse prevention. Techniques specifically targeting mood management, including those focused on anger and stress, were also employed. One study highlighted the potential effectiveness of a CBT-based smoking reduction program that integrates various clinical strategies.

### 3.6. Characteristics of the Sessions

Our systematic review revealed a wide range of techniques, each with differences in duration, style of intervention, and the impacts observed in the corresponding studies. In Table 5, we carefully highlight the key aspects that stand out as particularly relevant to each therapeutic approach.

### 3.7. Evaluators of the Results

During the systematic review conducted, a recurring problem was observed in many of the studies: a lack of clear information about the outcome assessors, which made it difficult to assess the risk of bias and discussions about blinding in the study process. In addition, a significant challenge highlighted in these experiments was a high dropout rate among participants. This dropout appears to be associated with difficulties experienced during abstinence and other symptoms that arise in the context of smoking cessation efforts [44,61].

## 4. Discussion

Systematic review of the scientific literature shows that educational processes on habituation and the implications of smoking, including its effects over different time periods and its important health consequences, often fail to have a significant impact on smokers [38,56].

It is important to consider that smoking represents a repetitive action that can become almost automatic, which complicates the smoking cessation process and the need to address it in a comprehensive manner [47]. In addition to the act of smoking itself, various environmental factors, such as the taste and smell of cigarettes, the sensation of holding a cigarette, and the longevity of these habits, often link smoking to positive experiences in the mind of the smoker [45].

From the first contact of nicotine with neurons, an arousal state is triggered that neurons remember, adapting their responses to subsequent nicotine exposure. This interaction leads to a dependence characterized by the neurons’ desire to re-experience this state of arousal [53]. As a result, this systematic review evidences a high dropout rate among participants in these studies, often due to difficulties associated with withdrawal and other symptoms related to the effects of nicotine [47].

The systematic review showed the most frequent limitations experienced when developing controlled clinical trials (RCTs), including clarity in the identification of therapists and evaluators, as well as transparency in blinding processes. This weakness impacts current smoking cessation strategies that minimal outcomes, such as occasional smoking or sporadic abstinence, have negligible effects on overall health outcomes, such as mortality and morbidity [44,46]. Restrictions on how RCTs are reported affected the review process, impacting the selection of studies was the occasional lack of clarity or omission of critical information in titles and abstracts, such as types of treatment, a situation that could cause confusion or misinterpretations of results.

Following this, it is important to critically evaluate the quality and generalizability of studies supporting smoking cessation recommendations and interventions [64,65]. In the RCTs studied, it was found that the psychological therapies and psychological approaches most implemented in controlled trials are cognitive behavioral therapy (CBT), mindfulness and cognitive behavioral therapy (CBT), behavioral therapy and psychosocial support, and other treatments, with financial incentives, video games, and combinations of procedures. It is worth highlighting that a relevant aspect of this review is the influence of participants’ willingness or determination to quit smoking on their commitment to treatment and persistence at follow-up [45]. 

Most of the studies, were based on psychological treatments aimed at reducing withdrawal symptoms in habitual cigarette users and performed pre- and post-intervention measurements through different means of verification with biomarkers (expired CO) or biochemically in blood and saliva sampling; however, the studies do not report results on long-term abstinence. Authors such as Jhanjee, Lal, Mishra [58] and Zakharova and Ibatov [56] reported variable abstinence rates depending on the type of treatment and duration of follow-up.

In the case of interventions with cognitive behavioral therapy (CBT), abstinence was maintained until the fourth week, with 22% in the PTSD-PTSD group and 13% in the non-PTSD group [60]. In turn, Laude et al. [46] compared prolonged versus nonprolonged CBT for smoking cessation. No significant differences in abstinence were observed at 52 and 104 weeks [46].

Combined treatments refer use of cognitive behavioral therapy—CBT, with mindfulness [48], home visits [62], and psychosocial support and telephone counseling [38]. Interventions appear to have positive effects on smoking cessation, with abstinence rates above 50% at 6 months [57]. Frings et al. [40] compared the Easyway program with a specialized pharmacological and behavioral support service; the combination of behavioral therapy and pharmacotherapy, in particular NRT and varenicline, are effective in promoting smoking cessation [66].

Regarding the use of financial incentives, its efficacy is presented in decreasing the severity of withdrawal symptoms [37] in time no longer than one-year post-intervention [47]. Video-based interventions such as those conducted by Bloom et al. [50] QuitBet platform, and Scholten et al. [49] with the go/no-go training game for smoking cessation in young adults. Neither baseline nor endpoint measures were reported [49].

Comparing the selected studies, it was found that cognitive behavioral therapy (CBT) is the most widely used method to reduce tobacco consumption. It is relevant to note that CBT encompasses a variety of techniques with significant efficacy in the reviewed studies. The most needed effects that were not addressed in the reviewed experiments are the rate of reduction in cigarette consumption, health-related quality of life, severity and duration of withdrawal symptoms, self-efficacy to quit, relapse, changes in attitudes and beliefs about smoking, and adherence to smoking cessation treatment [67].

The studies reviewed in this systematic review emphasize the immediate benefits of smoking cessation interventions, but also highlight a key concern: the long-term efficacy of these treatments remains uncertain, as the sustainability of abstinence and the possibility of relapse are major challenges [20]. Follow-up periods in these studies vary, ranging from brief check-ups to more comprehensive and continuous follow-up that includes in-person and telephone interactions, as well as various medical assessments. One study in particular extended the duration of its follow-up, providing a more robust dataset and underlining the importance of long-term observation for more reliable results, but aspects such as self-efficacy to quit, health-related quality of life, and improved family relationships are aspects that could increase motivation to quit [68].

## 5. Conclusions

In our quest to answer the central question of our systematic review—“What psychological therapies are effective in reducing smoking?”—we embarked on a thorough examination of relevant literature. Our search spanned six esteemed scientific bibliographic databases, leading to the selection of 28 studies that not only aligned with our precise inclusion criteria, but also passed the rigorous assessment based on the Joanna Briggs Institute checklists [36] (see Supplementary Material). Our systematic review methodology was meticulously aligned with the standards set by the PRISMA 2020 Declaration, an essential guide for the publication of systematic reviews [34].

The results obtained from the analysis revealed the existing psychological treatments which address smoking and also contribute to treatment adherence to be cognitive behavioral treatments, interventions that implement the use of mobile technology, mindfulness, and brief and motivational interventions. The psychological treatment that has shown efficacy in reducing smoking withdrawal symptoms is CBT, which is characterized by its structured approach and its focus on modifying cognitive and behavioral patterns associated with smoking.

The evidence gathered suggests that it is important to address smoking abstinence through preventive interventions, namely effective and personalized treatments that provide individuals with tools and resources that are tailored to their particular needs, promote well-being, enhance the quality of life of individuals, and attenuate the burden of tobacco-associated diseases.

## Figures and Tables

**Table 1:** Thesaurus terms.

Source	Keywords	Related Terms
DECS	Cigarette	Cigar, cigarette, cigarettes, cigars.
DECS	Smoking	Smokers, cigarette smoker, cigarette smokers.
DECS	Therapy	Therapeutic, therapy(s), treatment(s).
MESH	Nicotine Dependence	No records found.
MESH	Psychological Intervention	Psychological intervention, psychological interventions.
MESH	Smoking	No records found.
MESH	Psychological therapy	No records found.

**Table 2:** Databases with the applied search query.

Database	Search Algorithm
COCHRANE	(“Psychotherapy”) OR (“Psychological Treatment”) AND (“Tobacco”) OR (“Smoker”) OR (“Nicotine”)
PROQUEST	(“Psychotherapy”) OR (“Psychological Treatment”) AND (“Tobacco”) OR (“Smoker”) OR (“Smokers”) OR (“Nicotine”)
PSYCNET	(“Psychotherapy”) OR (“Psychological Treatment”) AND (“Tobacco”) OR (“Smoker”) OR (“Nicotine”)
PUBMED	(“Psychotherapy”) OR (“Psychological Treatment”) OR (“Intervention”) AND (“Tobacco”) OR (“Smoking”) OR (“Cigarette”) OR (“Cigar”) OR (“Smoker”) OR (“Smokers”) OR (“Nicotine”) AND (“Dependence”)
SCIENCE DIRECT	(“Psychotherapy”) OR (“Clinical Psychology”) OR (“Psychological Therapies”) AND (“Smoking”) OR (“Tobacco”) OR (“Smoker”) OR (“Cigar”) OR (“Cigarette”) OR (“Nicotine”)
SCOPUS	(“Psychotherapy”) OR (“Clinical Psychology”) OR (“Psychological Therapies”) AND (“Smoking”) OR (“Tobacco”) OR (“Smoker”) OR (“Cigar”) OR (“Cigarette”) OR (“Nicotine”)

**Table 3:** Filters applied.

Database	Total Number of Studies Found	Type of Document	Period	Incomplete/ Duplicated Texts	No Access	Noncompliance with Criteria	Total Number of Selected Studies
PROQ UEST	3,158,562	2,429,719	466,408	42,598	122,704	10,062	2
SCIEN CE DIREC T	1,333,687	563,134	586,014	142,449	38,747	3380	1
PUBM ED	425,914	409,030	13,610	1187	0	2084	8
COCH RANE	105,049	12,323	57,973	27,878	6387	834	0
SCOP US	25,276	4712	12,478	3047	3	4990	11
PSYC NET	125,895	79,694	59,116	7607	0	2577	6
TOTAL	5,174,383	3,498,612	1,195,599	224,766	167,841	23,927	28

**Table 4:** Selected studies.

Study	Database	Title	Author(s)	Year
1	PROQUEST	Financial incentives to Medicaid smokers for engaging tobacco quit line treatment: maximizing return on investment	Mundt, Baker, Piper, Smith, Fraser, and Fiore [37].	2020
2	PROQUEST	Cost-effectiveness analysis of smoking cessation interventions using cell phones in a low-income Population	Daly, Deshmukh, Vidrine, Prokhorov, Tahaky, Houchen, and Cantor [38].	2019
3	SCIENCE DIRECT	Treatment completion and anxiety sensitivity effects on smoking cessation outcomes	Martínez, López, Rodríguez, Senra, and Becoña [39].	2021
4	PUBMED	Smoking cessation intervention for severe mental ill health trial (SCIMITAR+): study protocol for a randomized controlled trial	Peckham, Arundel, Bai ley, Brownings, Fairhurst, Heron, Li, Parrott, and Gilbody [40].	2017
5	PUBMED	Comparison of Allen Carr’s Easy way programmed with a specialist behavioral and pharmacological smoking cessation support service: a randomized controlled trial	Frings, Albery, Moss, Brunger, Burghelea, White, and Wood [41].	2020
6	PUBMED	A pilot randomized clinical trial of brief interventions to encourage quit attempts in smokers from socioeconomic disadvantage	Steinberg, Rosen, Versella, Borges, and Leyro [42].	2020
7	PUBMED	Effectiveness of village health worker-delivered smoking cessation counseling in Vietnam	Jiang Siman, Cleland, Van, Nguyen, Nguyen, and Shelley [43].	2019
8	PUBMED	The effects of mindfulness- based yogic breathing on craving, affect and smoking behavior	Lotfalian, Spears, and Juliano [31].	2020
9	PUBMED	Heterogeneous treatment effects of a text messaging smoking cessation intervention among university students	Bendtsen [44].	2020
10	PUBMED	Mechanisms underlying mindfulness- based addiction treatment versus cognitive behavioral therapy and usual care for smoking Cessation	Spears, Hedeker, Li, Wu, Anderson, Houchins, Vinci, Hoover, Vidrine, Cinciripini, Waters, and Wetter [45].	2017
11	PUBMED	Extended treatment for cigarette smoking cessation: a randomized control trial	Laude, Bailey, Crew, Varady, Lembke, McFall, Jeon, Killen, Killen, and David [46].	2017
12	PSYCNET	Feasibility, tolerability, and potential advantages of a dyadic financial incentive treatment for smoking cessation among dual-Smoker couples: a pilot study	Haskins, Payne, Schiavone, Beach, MacKillop, and VanDellen [47].	2021
13	PSYCNET	A pilot randomized controlled trial of smartphone- assisted mindfulness- based intervention with contingency management for smokers with mood disorders	Minami, Nahvi, Arnsten, Brinkman, Rivera, Wetter, Bloom, Price, Richman, Betzler, Stockmal, Donnelly, McClain, Kennedy, Vieira, Fine, McCarthy, Thomas, Hecht, and Brown [48].	2021
14	PSYCNET	Mechanisms of change in a Go/No-Go training game for young adult smokers	Scholten, Hanneke Luijten, Maartje Poppelaars, Anouk Johnson-Glenberg, Granic, Isabela [49].	2021
15	PSYCNET	Pilot trial of Quit Bet: A digital social game that pays you to stop smoking	Bloom, Japuntich, Pierro, Dallery, Leahey, and Rosen [50].	2021
16	PSYCNET	Effects of a brief motivational smoking intervention in non-treatment seeking disadvantaged Black smokers	Brett, Chavarria, Liu, Hedeker, and King [28].	2021
17	PSYCNET	Increasing cessation motivation and treatment engagement among smokers in pain: A pilot randomized controlled trial	Zale, Maisto, De Vita, Hooten, and Ditre [51].	2021
18	SCOPUS	Evaluation of the effectiveness of a WHO5A model based comprehensive tobacco control program among migrant workers in Guangdong China: a pilot study	Chai, Zou, Shi, Chen, Gong, Wei, and Ling [52].	2018
19	SCOPUS	Smoking cessation in workplace settings: quit rates and determinants in a group behavior therapy programmed	Hausherr, Quinto, Grize, Schindler, and Probst [53].	2017
20	SCOPUS	Physical activity and quit motivation moderators of adolescent smoking reduction	Blank, Ferris, Metzger, Gentzler, Duncan, Jarrett, and Dino [54].	2017
21	SCOPUS	A telephone- based guided imagery tobacco cessation intervention: results of a randomized feasibility trial	Gordon, Bell, Armin, Giacobbi, and Nair [55].	2021
22	SCOPUS	Effectiveness of drug and non- drug treatment of tobacco dependence among medical Workers	Zakharova and Ibatov [56].	2021
23	SCOPUS	Combined treatment for at- risk drinking and smoking cessation among Puerto Ricans: A randomized clinical trial	Correa, Díaz, Reitzel, Guo, Chen, Li, Calo, Shih YT, and Wetter DW [57].	2017
24	SCOPUS	A randomized pilot study of brief intervention versus simple advice for women tobacco users in an urban community in India	Jhanjee, Lal, Mishra, and Yadav [58].	2017
25	SCOPUS	A randomized controlled trial of distress tolerance treatment for smoking cessation	Brown, Palm, Bloom, Minami, Strong, Lejuez, Zvolensky, and Hayes [59].	2018
26	SCOPUS	Contingency management and cognitive behavioral therapy for trauma-exposed smokers with and without posttraumatic stress disorder	Japuntich, Lee, Pineles, Gregor, Joos, Patton, Krishnan, and Rasmusson [60].	2019
27	SCOPUS	A randomized controlled trial of inhibitory control training for smoking cessation and reduction	Bos, Staiger Hayden, Hughes, Youssef, and Lawrence [61].	2019
28	SCOPUS	Combination extended smoking cessation treatment plus home visits for smokers with schizophrenia: A randomized controlled trial	Brody, Zorick, Hubert, Hellemann, Balali, Kawasaki, Garcia, Enoki, Abraham, Young, and McCreary [62].	2017

**Table 5:** Intervention characteristics.

No.	Study	Authors	Year	Treatment	Measurements Made in the Study	
					Initial	Finale
1	Financial incentives to Medicaid smokers for engaging tobacco quit line treatment: maximizing return on investment [37].	Mundt, Baker, Piper, Smith, Fraser, and Fiore	2020	Incentive (USD 30) Wisconsin tobacco Quitline (WTQL)	Biochemically confirmed 7-day point abstinence at the 6-month follow-up visit.	21.6% of the participants in the incentive group were biochemically confirmed as abstinent at 6-month follow-up vs. 13.7% in the control group.
2	Cost-effectiveness analysis of smoking cessation interventions using cell phones in a low-income population [38].	Daly, Deshmukh, Prokhorov, Houchen, and Cantor	2019	No report	11 telephone counseling sessions were scheduled during the 12-week treatment period. The first session took place one day before the quit date, the next four sessions were scheduled during the first week after quitting, and the remaining six sessions were scheduled every two weeks until the end of treatment.	All active participants were followed for 6 months after enrollment and were asked how their smoking habits had changed by cell phone assessments.
3	Effects of treatment completion and anxiety sensitivity on smoking cessation outcomes [39].	Martínez-Vispo, López-Duran, Rodríguez-Cano, Senra and Becoña	2021	Standard cognitive-behavioral smoking cessation treatment (SCBSCT). Standard cognitive-behavioral smoking cessation treatment with behavioral activation components (SCBSCT-BA). (c) Waiting list control group.	No report	Participants were considered abstainers if they reported abstinence, not even a puff, for ≥30 days at 3-, 6-, and 12-month follow-up, and had an expired carbon monoxide (CO) reading of <6 parts per million.
4	Smoking cessation intervention for severe mental health trial (SCIMITAR +): study protocol for a randomized controlled trial [40].	Peckham, Arundel, Bailey, Brownings, Fairhurst, Heron, Li, Parrott, and Gilbody	2017	Hypnotic suggestion condition Stroop test	No report	The primary outcome will be self-reported smoking cessation at 12 months, verified by measurement of expired carbon monoxide (CO).
5	Comparison of Allen Carr’s Easy way program with a specialized pharmacological and behavioral support service for smoking cessation: a randomized controlled trial [41].	Frings, Albery, Moss, Brunger, Burghelea, White and Wood	2020	Allen Carr’s Easy way (ACE) Specialized behavioral and pharmacological support	No report	The primary outcome was self-reported continuous abstinence for 26 weeks from the quit/smoking cessation reset date verified by a measurement of exhaled breath carbon monoxide <10 parts per million (ppm). The primary analysis was by intention-to-treat. Secondary outcomes were use of pharmacotherapy, adverse events, and continued abstinence up to 4 and 12 weeks.
6	A pilot randomized clinical trial of brief interventions to encourage quit attempts among socioeconomically disadvantaged smokers [42].	Steinberg, Rosen, Versella, Borges and Leyro	2020	Nicotine replacement therapy. Motivational interviewing	No report	Follow-up was completed approximately 1 month after the intervention.
7	Effectiveness of smoking cessation counseling delivered by village health workers in Vietnam [43].	Jiang, Siman, Cleland, Van Devanter, Nguyen, Nguyen, and Shelley	2019	Counseling and assistance from the health care provider (ARM 1). ARM 1 plus advice from village health workers (VHWS) (ARM 2).	The main outcome of this study was the point prevalence of 7 days	At 6-month follow-up, abstinence rates in ARM 2 were significantly higher than those in ARM 1 (25.7% vs. 10.5%; *p* < 0.001).
8	The effects of mindfulness-based yogic breathing on craving, affect, and smoking [31].	Lotfalian, Spears and Juliano	2020	Yogic breathing intervention (MB). Active treatment (cognitive strategy [CS]). No treatment (NT)	No report	No report
9	Heterogeneous treatment effects of a text-messaging smoking cessation intervention among college students [44].	Bendtsen	2020	Smoking cessation interventions via text messaging.	No report	At 3 months after randomization, follow-up, data were collected from 1502 students (94.5%, 1502/1590). The primary outcome measure in the next trial was subjective reporting of prolonged abstinence, following Russel’s standard definition [20], as not having smoked more than 5 cigarettes in the past 8 weeks.
10	Mechanisms underlying mindfulness-based addiction treatment versus cognitive behavioral therapy and usual care for smoking cessation [44].	Spears, Hedeker, Li, Wu, Anderson, Houchins, Vinci, Hoover, Vidrine, Cinciripini, Waters and Wetter	2017	Mindfulness-based addiction treatment (MBAT). Cognitive behavioral therapy (CBT). Habitual Care (UC) for smoking cessation (all participants received self-help materials based on the Clinical Practice Guideline for the Treatment of Tobacco Use and Dependence (Fiore et al., 2008), psychoeducation on tobacco dependence/relapse/relapse and nicotine patch therapy).	Biochemically confirmed 7-day abstinence from smoking	4 and 26 weeks after quitting smoking.
11	Extended treatment for cigarette smoking cessation: a randomized control trial [46].	Laude, Bailey, Crew, Varady, Lembke, McFall, Jeon, Killen, Killen, and David	2017	Prolonged cognitive behavioral therapy. Nonprolonged cognitive behavioral therapy.	The primary outcome was the 7-day point prevalence (PP) confirmed by expired CO	PP abstinence rates at 52-week follow-up were comparable between the nonextended CBT (40%) and CBT-E (39%) groups [odds ratio (OR) = 0.99; 95% confidence interval (CI) = 0.55, 1.78]. A similar pattern was observed in the nonextended CBT (39%) and CBT-E (33%) groups at 104-week follow-up (OR = 0.79; 95% CI = 0.44, 1.40).
12	Feasibility, tolerability, and potential advantages of a dyadic financial incentive treatment for smoking cessation among dual-smoking couples [47].	Haskins, Payne, Schiavone, Beach, MacKillop and VanDellen	2021	Financial incentive treatments	Among participants who completed the follow-up session, cravings for smoking and the severity of tobacco withdrawal symptoms were reduced during the study period.	No report
13	A pilot randomized controlled trial of smartphone-assisted mindfulness-based intervention with contingency management for smokers with mood disorders [48].	Minami, Navhi, Arnesten, Brikman, Rivera-Mindt, Wetter, Bloom, Price, Richman, Betzler, Stockmal, Donnelley, McClain, Kennedy, Viera, Fine, McCarthy, Thomas, Hecht, and Brown	2021	Mindfulness intervention	Biochemically verified 7-day point prevalence abstinence	2, 4 y 13 weeks
14	Mechanisms of change in a Go/No-Go training game for young adult smokers [49].	Scholten, Luijten, Poppelaars, Johnson-Glenberg and Granic	2021	Hitnrun	No report	No report
15	Quit Bet pilot test: a digital social game that pays you to quit smoking [50].	Bloom, Japuntich, Pierro, Dallery, Leahey and Rosen	2021	Didactical quit bet	After a week to prepare for quitting, quit day was day 8. Between day 9 and day 28 (a 20-day period), participants recouped USD 1 of their USD 30 wager for each day of verified abstinence with carbon monoxide (co) (≤6 ppm). The remaining stake money was combined into a “grand prize” pot. Participants who were abstinent on at least 19 out of the 20 days	No report
16	Effects of a brief motivational intervention on smoking in disadvantaged black smokers who do not seek treatment [28].	Brett, Chavarria, Liu, Hedeker and King	2021	Motivational intervention	No report	No report
17	Increased motivation to quit smoking and commitment to treatment among smokers with pain: a randomized controlled pilot trial [51].	Zale, Maisto, De Vita, Hooten and Ditre	2021	Motivational intervention psychoeducation about smoking	No report	At 1-month follow-up, advances in the knowledge of the interrelations between pain and smoking were maintained (*p* = 0.009).
18	Evaluating the effectiveness of a comprehensive tobacco control program based on the WHO-5A model among migrant workers in Guangdong, China: a pilot study [52].	Chai, Zou, Shi, Chen, Gong, Wei, and Ling	2020	Model WHO-5A (OMS 5A)	No report	The primary outcome was the change in smoking rate according to salivary cotinine concentration at 3-month follow-up compared to the control arm.
19	Smoking cessation in the workplace: quit rates and determinants in a group behavioral therapy program [53].	Hausherr, Quinto, Grize, Schindler and Probst	2018	Cognitive behavioral therapy. Cognitive preparation, motivation, psychoeducation. Motivation, reinforcement of ambivalence, self-control. Motivation, psychoeducation, coping skills, behavioral alternatives to smoking. Coping skills, self-management. Proactive telephone counseling	The evaluation consisted of three anonymized questionnaires (pre- and post-intervention and 12-month follow-up).	The evaluation consisted of three anonymized questionnaires (pre- and post-intervention and 12-month follow-up).
20	Moderators of physical activity and motivation to quit smoking in reducing adolescent smoking [54].	Blank, Ferris, Metzger, Gentzler, Duncan, Jarrett, and Dino	2017	Brief motivational intervention with promotion of healthy habits emphasizing physical activity.	They were significantly correlated at both baseline and 3-month follow-up.	Were significantly correlated at both baseline and correlated at both baseline and 3-month follow-up.
21	A telephone smoking cessation intervention with guided imagery: results of a randomized controlled trial [55].	Gordon, Bell, Armin, Giacobbi, and Nair	2017	Intervention with guided imagery and active behavioral control	Not specified, however it can be interpreted that measurement was generated at the end of treatment.	Ee evaluate 6-month dropout rates
22	The efficacy of pharmacological and nonpharmacological treatment of tobacco dependence among health professionals [56].	Zakharova and Ibatov	2020	Cognitive behavioral therapy, psychosocial support using cognitive aspects; brief psychotherapy; breathing exercises; acupuncture and increased physical activity.	After treatment in the second group, 195 (64%) people out of 305 people stopped smoking completely, in the first group of medical workers who received nondrug therapy, 177 (56%) people out of 316 people stopped smoking completely.	6 months after the end of the treatment program, 26.7% (84 medical workers) returned to smoking in group 1 and 10.2% (31 medical workers) in group 2 (or 3.02, 95% ci 2, 05–5.02; *p* < 0.00001).
23	Combination treatment for smoking cessation and at-risk alcohol use among Puerto Ricans: a randomized clinical trial [57].	Correa, Díaz, Reitzel, Guo, Chen, Li, Calo, Shih and Wetter	2020	Behavioral intervention, motivation, and problem solving.	Blinded follow-up evaluations were performed by telephone at weeks 12, 26, and 52.	Blinded follow-up evaluations were performed by telephone at weeks 12, 26, and 52.
24	A Randomized Pilot Study of Brief Intervention versus Counseling of Brief Intervention versus Simple Counseling for Women Tobacco Users in an Urban Indian Community. urban community in India [58].	Jhanjee, Lal, Mishra, and Yadav	2017	Brief psychoeducation and/or counseling intervention	All participants were tracked and evaluated at one week.	3 months after surgery
25	A randomized controlled trial of stress tolerance treatment for smoking cessation [59].	Brown, Palm, Bloom, Minami, Strong, Lejuez, Zvolensky and Hayes	2017	Standard behavioral protocol (Brown, 2003). Distress tolerance treatment.	There was no significant difference between conditions in the primary outcome of biochemically verified 7-day point prevalence smoking abstinence after 7 days.	No report
26	Contingency management and cognitive behavioral therapy for trauma-exposed smokers with and without posttraumatic stress disorder [60].	Japuntich, Lee, Pineles, Gregor, Joos, Patton, Krishnan and Rasmusson	2018	Cognitive behavioral therapy	Seven-day post-quit abstinence rates for participants with and without PTSD, respectively, were similar: 39% vs. 38% (1-week), 33% vs. 28% (2-week).	Abstinence rates, 22% vs. 19% (3 weeks) and 22% vs. 13% (4 weeks).
27	A randomized controlled trial of inhibitory control training for smoking cessation and reduction [61].	Bos, Staiger Hayden, Hughes, Youssef, and Lawrence	2019	Inhibitory control training. Go/no-go training.	No report	No report
28	Prolonged combined smoking cessation treatment plus home visits for smokers with schizophrenia: randomized controlled trial [62].	Brody, Zorick, Hubert, Hellemann, Balali, Kawasaki, Garcia, Enoki, Abraham, Young and McCreary	2017	Cognitive behavioral therapy	7-day point prevalence abstinence rates for the three groups were 45%, 20%, and 8%.	No report

## Data Availability

No new data were created or analyzed in this study.

## Supplementary Materials

The following supporting information can be downloaded at: https://www.mdpi.com/article/10.3390/ijerph21060753/s1, Attachment S1: JBI Critical Appraisal Checklist for systematic reviews and research syntheses.

## Abbreviations

ACE	Allen Carr’s Easy way
CAD	Coronary artery disease
CBT	Cognitive behavioral therapy
COPD	Chronic obstructive pulmonary disease
CS	Cognitive strategy
EC	Enhanced care.
EST	Enhanced standard treatment.
IC	Intensive care
MBAT	Mindfulness-based addiction treatment
MESH	Medical Subject Headings
NRT	Nicotine replacement therapy
NT	No treatment
SC	Standard Care
TAU	Treatment as usual
UC	Usual care
PHS	Public Health Service
MB	Mindfulness-based yogic breathing
CBT	Cognitive behavioral therapy
PP	Point-prevalence
DSC	Dual-smoker couples
N-O-T	Not-on-Tobacco
PA	Physical activity
N-O-T+FIT	Module
BI	Brief Intervention
CI	Intervention Condition
