# Supplementary material for: Psychological Therapies Used for the Reduction of Habitual Cigarette Smoking Cigarette Consumption: A Systematic Review
Source: Int J Environ Res Public Health. 2024 Jun 9;21(6):753. doi: 10.3390/ijerph21060753 (PMC11203857; doi:10.3390/ijerph21060753)
Supplement: Supplementary file 1 [file ijerph-21-00753-s001.zip › ijerph-2868583-supplementary.pdf]

Supplementary Material

Psychological Therapies Used for the Reduction of Habitual Cigarette Smoking Cigarette Consumption: A Systematic Review

Sandra-Milena Carrillo-Sierra [1,*], Lorena Cárdenas-Cáceres [1], John Anderson Cadrazco-Urquijo [1], Angie Natalia Salazar-Gómez [1], Diego Rivera-Porras [1] and Valmore Bermúdez [2]

[1] Facultad de Ciencias Jurídicas y Sociales, Centro de Investigación en Estudios Fronterizos, Universidad Simón Bolívar, Cúcuta 540001, Colombia; l_cardenas1@unisimon.edu.co (L.C.-C.); j_cadrazco@unisimon.edu.co (J.A.C.-U.); a_salazar7@unisimon.edu.co (A.N.S.-G.); diego.rivera@unisimon.edu.co (D.R.-P.)

[2] Facultad de Ciencias de la Salud, Centro de Investigaciones en Ciencias de la Vida, Universidad Simón Bolívar, Barranquilla 080001, Colombia; valmore.bermudez@unisimon.edu.co

* Correspondence: sandra.carrillo@unisimon.edu.co

Attachment 1. JBI Critical Appraisal Checklist for systematic reviews and research syntheses.

JBI CRITICAL APPRAISAL CHECKLIST FOR SYSTEMATIC REVIEWS AND RESEARCH SYNTHESES

Reviewer_______________________________ Date_________________________

Author_______________________________ Year_______ Record Number_________

	Yes	No	Unclear	Not applicable
1. Is the review question clearly and explicitly stated?	☐	☐	☐	☐
2. Were the inclusion criteria appropriate for the review question?	☐	☐	☐	☐
3. Was the search strategy appropriate?	☐	☐	☐	☐
4. Were the sources and resources used to search for studies adequate?	☐	☐	☐	☐
5. Were the criteria for appraising studies appropriate?	☐	☐	☐	☐
6. Was critical appraisal conducted by two or more reviewers independently?	☐	☐	☐	☐
7. Were there methods to minimize errors in data extraction?	☐	☐	☐	☐
8. Were the methods used to combine studies appropriate?	☐	☐	☐	☐
9. Was the likelihood of publication bias assessed?	☐	☐	☐	☐
10. Were recommendations for policy and/or practice supported by the reported data?	☐	☐	☐	☐
11. Were the specific directives for new research appropriate?	☐	☐	☐	☐

Overall appraisal: Include ☐ Exclude ☐ Seek further info ☐

Comments (Including reason for exclusion)

____

____
